# Towards an in vitro fibrogenesis model of human vocal fold scarring

**DOI:** 10.1007/s00405-018-4922-7

**Published:** 2018-03-08

**Authors:** M. Graupp, B. Rinner, M. T. Frisch, G. Weiss, J. Fuchs, M. Sundl, A. El-Heliebi, G. Moser, L. P. Kamolz, M. Karbiener, M. Gugatschka

**Affiliations:** 1 Department of Phoniatrics, ENT University Hospital Graz, Medical University of Graz, Auenbruggerplatz 26, 8036 Graz, Austria; 2 Division of Biomedical Research, Core Facility Alternative Biomodels and Preclinical Imaging, Medical University of Graz, Graz, Austria; 3 Institute of Cell Biology, Histology and Embryology, Medical University of Graz, Graz, Austria; 4 Division of Plastic, Aesthetic and Reconstructive Surgery, Department of Surgery, Medical University of Graz, Graz, Austria

**Keywords:** Vocal fold fibroblasts, Vocal fold scar, In vitro fibrogenesis model, Macromolecular crowding

## Abstract

**Background:**

Vocal fold (VF) scarring remains a therapeutic dilemma and challenge in modern laryngology. To facilitate corresponding research, we aimed to establish an in vitro fibrogenesis model employing human VF fibroblasts (hVFF) and the principles of macromolecular crowding (MMC).

**Methods:**

Fibrogenesis was promoted by addition of transforming growth factor-β1 to standard medium and medium containing inert macromolecules (MMC). Hepatocyte growth factor (HGF) and Botox type A were tested for their antifibrotic properties in various doses. Experiments were analyzed with respect to the biosynthesis of collagen, fibronectin, and α-smooth muscle actin using immunofluorescence, silver stain and western blot.

**Results:**

MMC led to favourable enhanced deposition of collagen and other extracellular matrix components, reflecting fibrotic conditions. Low doses of HGF were able to dampen profibrotic effects. This could not be observed for higher HGF concentrations. Botox type A did not show any effects.

**Conclusion:**

Based on the principles of MMC we could successfully establish a laryngeal fibrogenesis model employing hVFF. Our finding of dose-dependent HGF effects is important before going into clinical trials in humans and has never been shown before. Our model provides a novel option to screen various potential antifibrotic compounds under standardized conditions in a short time.

**Electronic supplementary material:**

The online version of this article (10.1007/s00405-018-4922-7) contains supplementary material, which is available to authorized users.

## Introduction

Vocal fold (VF) scarring remains one of the most difficult to treat conditions among benign VF diseases. Main features of VF scarring are disorganized collagen and elastin bundles, loss of important extracellular matrix (ECM) constituents, volume deficiency, reduced VF pliability and glottal insufficiency [1]. Various surgical procedures have been developed to treat this condition but none could fully restore vibration. Also transplantation of similar, autologous tissues (oral mucosa) cannot rebuild the specific micro-architecture [2].

Newer therapeutic approaches aim to restore function on a cellular basis and can be summarized under the term laryngeal tissue engineering [3]. A decisive factor for VF healing and thereby for laryngeal tissue engineering are the site-specific VF fibroblasts (VFF), which are responsible for synthesis of interstitial proteins (e.g. fibronectin), glycosaminoglycans [e.g. hyaluronic acid (HA)] and ECM fibers (e.g. different types of collagens, elastin) [4]. During fibrogenesis, VFF transforms into myofibroblasts (scar fibroblasts) with a markedly different biological behaviour and ECM production profile [5]. Using VFF in a reliable in vitro model of VF scarring and fibrogenesis might be a promising approach in fibrosis research, as it would allow targeting the fibrotic cascade at different stages that are otherwise not accessible.

Different fibrogenesis models were proposed, but many of them are characterized by a slow in vitro procollagen—and hence, collagen-matrix deposition [6, 7]. This is due to the transfer of cells from a context of highly dense arrays of macromolecules, the ECM, into culture plastic plates with an aqueous medium. Adding inert macromolecules to the cell culture medium (“crowding”) the in vitro collagen deposition can be upregulated significantly [8]. The underlying mechanism is based upon the excluded volume effect (EVE). Upon macromolecular crowding (MMC), the added macromolecules occupy a substantial volume of the culture medium and thereby confine other molecules to the remaining space, which enhances reaction kinetics and molecular assembly [6].

We pursued a pilot study using rats’ VFF to establish a fibrogenesis model based on the principles of MMC [9]. The next step was to transfer these findings into the human setting by employing human VFF and to test different antifibrotic compounds.

## Materials and methods

### Cell culture

Immortalized human vocal fold fibroblasts (hVFF) were obtained from Thibeault [10]. Dulbecco’s modified Eagle’s medium (DMEM) (Life Technologies, Gaithersburg, MD) supplemented with 10% fetal calf serum (FCS; Sigma-Aldrich St. Louis, MO, USA) and 1% penicillin/streptomycin (P/S) was used as standard growth medium. All cells were kept in a humidified 5% CO_2_ atmosphere at 37 °C. Experiments were performed with four replicates.

Cells foreseen for SDS-PAGE and western blot were seeded into 12-well-plates (Nalgen Nunc International, NY, USA; 60,000 cells/well). For Immunofluorescence, cells were grown on 4-well glass chamber slides (Lab-Tek II, Nalgene Nunc International, Naperville, IL, USA; 25,500 cells/chamber). 24 h after seeding, growth medium was switched to FCS-free medium for another 24 h. Cells were then allocated to different treatment groups as follows: For the control group DMEM enriched with 0.5% FCS, 1% P/S, and 100 mM of l-ascorbic acid 2-phosphate [11] was used, this is also referred to as standard medium. To induce myofibroblast differentiation 5 ng/mL transforming growth factor beta-1 (TGFβ-1) (Sigma-Aldrich St. Louis, MO, USA) was added, as described earlier [12]. “Crowded” conditions were generated in another group by adding a mixture of 37.5 mg/mL 70 kDa Ficoll (Fc) (Sigma-Aldrich St. Louis, MO, USA) with 25 mg/mL 400 kDa Fc [13, 14]. Moreover, TGFβ-1 and macromolecules were added simultaneously in one group, representing the condition of maximal fibrosis [13].

To test antifibrotic compounds, hepatocyte growth factor (HGF) (Sigma-Aldrich St. Louis, MO, USA) and Botox type A (BTXA; IPSEN Pharma, Germany) were added in different concentrations to groups with TGFβ-1 and macromolecules. HGF has been studied for its antifibrotic properties in various fields [15, 16] and was added at 40, 100 or 200 ng/mL. Botox is used in different clinical settings, among others for the treatment of hypertrophic scars [17] and was tested in our experiments at 40 or 80 IU/mL. After 5 days of incubation, samples were further processed.

### Immunofluorescence

hVFF were washed twice with phosphate buffered saline (PBS), fixed with methanol (− 20 °C), air dried for 30 min and stored at − 20 °C. 30 min prior to immunofluorescence staining cells were thawed and blocked with 3% BSA (Sigma Aldrich, St. Louis, MO, USA) for 1 h. Subsequently, cells were immune-labelled using the UltraVision LP Detection System (Thermo Scientific, Fremont, CA, USA). The following antibodies were diluted in antibody diluent (Dako, Glostrup, Denmark) and applied for 90 min at room temperature: ACTA-2 aka. α-Smooth muscle actin (α-SMA) (goat IgG, 20 mg/mL, LifeSpan BioSciences, Seattle, WA, USA); collagen-1 (mouse IgG, Dilution 1:1000, Sigma Aldrich, MO, USA, product no. C2456); fibronectin (rabbit IgG, 0.56 µg/mL, Proteintech, Chicago, IL, USA). Cells were washed three times with PBS followed by incubation with the secondary antibody (Alexa Fluor 555 Donkey Anti-goat IgG, 10 mg/mL; Alexa Fluor 488 Donkey Anti-mouse IgG, 10 mg/mL, Alexa Fluor 488 Donkey Anti-rabbit IgG, 10 mg/mL; all from Life Technologies, Carlsbad, CA, USA) and DAPI (Life Technologies, Carlsbad, CA, USA) for 45 min. Slides were washed again with PBS, mounted with ProLong Gold antifade reagent (Life Technologies, Gaithersburg, MD, USA) and observed with a Leica DM600B fluorescent microscope (Leica, Wetzlar, Germany) connected to an Olympus DP72 digital camera (Olympus, Tokyo, Japan).

### Cell count analysis after HGF treatment

For quantitative analysis of cells treated with HGF, chamber slides were stained with DAPI (Life Technologies, Carlsbad, CA, USA). A microscope (model DM6000B; Leica) equipped with a motorized stage and a digital camera (Olympus, Tokyo, Japan) was used for the acquisition of ten images per chamber. Images were randomly selected by the Visiopharm software (Hoersholm, Denmark) and cell nuclei were counted manually afterwards.

### Pepsin digestion, sodium dodecylsulphate-polyacrylamide gel electrophoresis (SDS-PAGE) and silver stain

Supernatants and cell layers from 12-well-plates were harvested separately and pepsinized as described before [18]. Briefly, 50 µL of a pepsin stock solution (1 mg/mL dissolved in 1N HCL; Roche Applied Sciences, Basel, SUI) were added to 500 µL of supernatants, while a pepsin digestion solution (25% pepsin stock solution, 0.005% Triton X-100 (Bio-Rad Laboratories, Hercules, CA, USA), 0.01% Phenol Red (Sigma-Aldrich, St. Louis, MO, USA) in ddH_2_O) was added to cell layers. All samples were incubated for 2 h on orbital shakers followed by neutralization with 1N NaOH.

SDS-PAGE was performed under non-reducing conditions using 3–8% precast Criterion XT Tris–Acetate gels (Bio-Rad Laboratories, Hercules, CA, USA) and XT Tricine running buffer (Bio-Rad Laboratories, Hercules, CA, USA); electrophoresis was run for 60 min at 200 V. VitroCol, human collagen I solution and human collagen solution type III (both Cell Systems, Troisdorf, Germany) served as collagen standards (0.16 µg/lane). Gels were subsequently stained with the SilverQuest™ Silver Staining kit (Thermo Fisher Scientific, Rockford, IL, USA) according to the manufacturer’s protocol. Gel images were acquired using Quantity One software (Bio-Rad Laboratories, Hercules, CA, USA), and densitometric analysis of bands was subsequently performed using ImageJ.

### Western blot

Proteins were extracted from the cell layer and subjected to SDS-PAGE using 4–12% Criterion XT Bis-Tris Gels (Biorad, Vienna, Austria). SDS-PAGE was run at 200 V for 60 min, followed by electroblotting of proteins (90 min at 0.5 A and 4 °C) onto Immobilon PVDF membranes (Millipore, MA, USA). Immuno-detection was carried out in Tris-buffered saline supplemented with 0.1% Tween-20 (Carl Roth, Karlsruhe, Germany) and 2.5% milk or 5% BSA. Membranes were incubated overnight at 4 °C with primary antibodies for detection of fibronectin (FN1; #15613, Proteintech), α-SMA (ACTA2; #LS-B3933, LSBio, WA, USA) and glyceraldehyde-3-phosphate dehydrogenase (GAPDH; #2118C, Cell Signaling, MA, USA). Subsequently, blots were incubated with secondary goat-anti-rabbit (DAKO, Vienna, Austria) or rabbit-anti-goat (DAKO) antibodies conjugated to HRP for 1 h at room temperature. The signal was detected by chemiluminescence. Densitometric analyses were conducted using ImageJ; band intensities of the proteins of interest were normalized to GAPDH. For statistical analyses signals were further normalized to the group treated with TGFβ-1 (NC/T).

### Statistical analysis

Differences of the mean were analyzed by paired t-tests using Predictive Analytics Software (PASW) statistics 21.0 (SPSS Inc., Chicago, IL, USA). 0.05 was chosen as a level for statistical significance.

## Results

### Validation of the in vitro fibrogenesis model

**Fig. 1:**
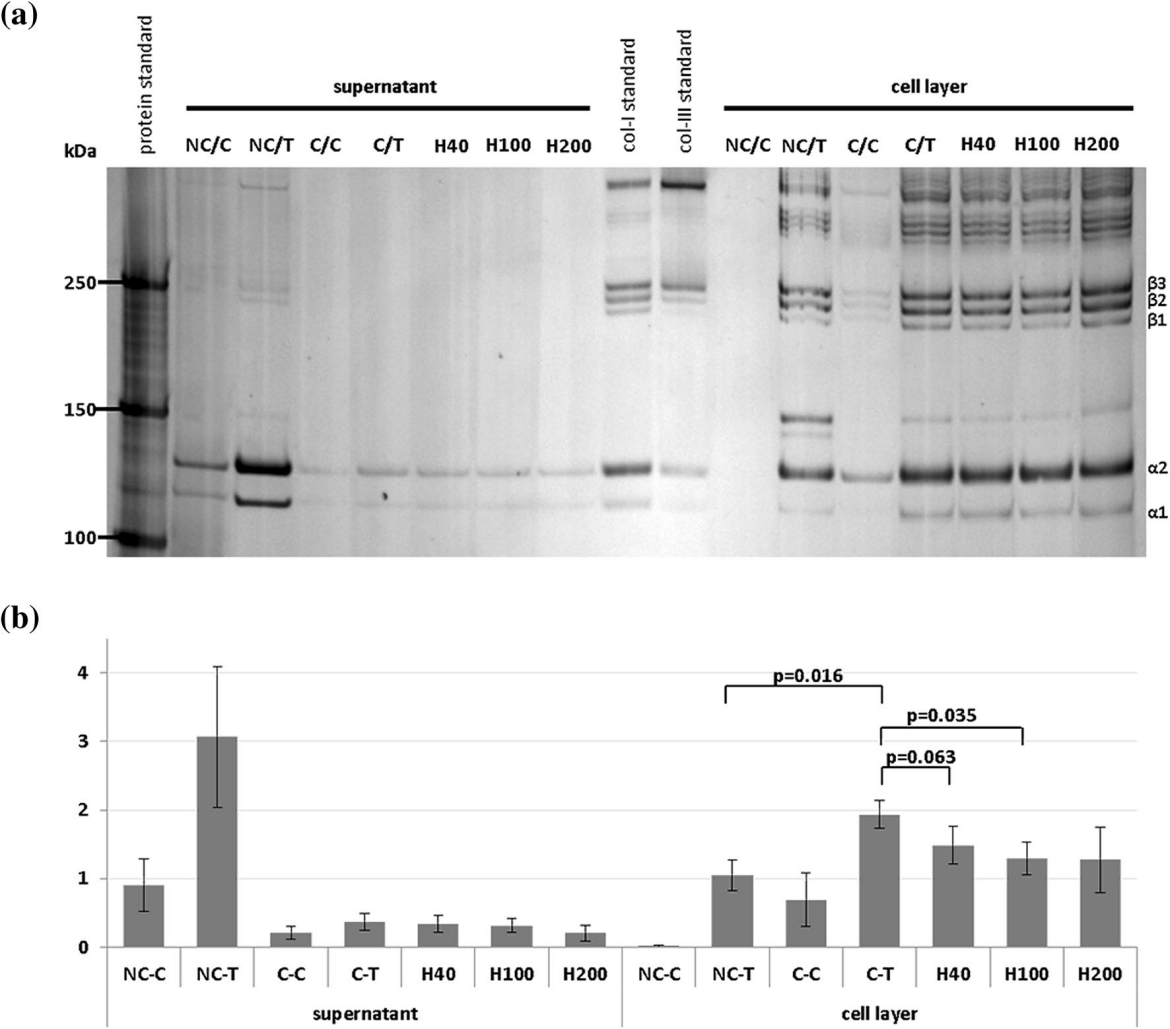
Effects of TGF-β1, macromolecular crowding and HGF on collagen biosynthesis analyzed by silver stain. (**a**) Image of silver stained gel showing results for collagen deposition in standard conditions and with HGF treatment; NC/C = non-crowded control; C/C = crowded control; NC/T = non-crowded + TGF-β1; C/T = crowded + TGF-β1; H40 = crowded + TGF-β1 + HGF 40 ng/ml; H100 = crowded + TGF-β1 + HGF 100 ng/ml; H200 = crowded + TGF-β1 + HGF 200 ng/ml; (**b**) Densitometric analysis of silver stain for collagen deposition; condition NC/C of cell layer served as reference

**Fig. 2:**
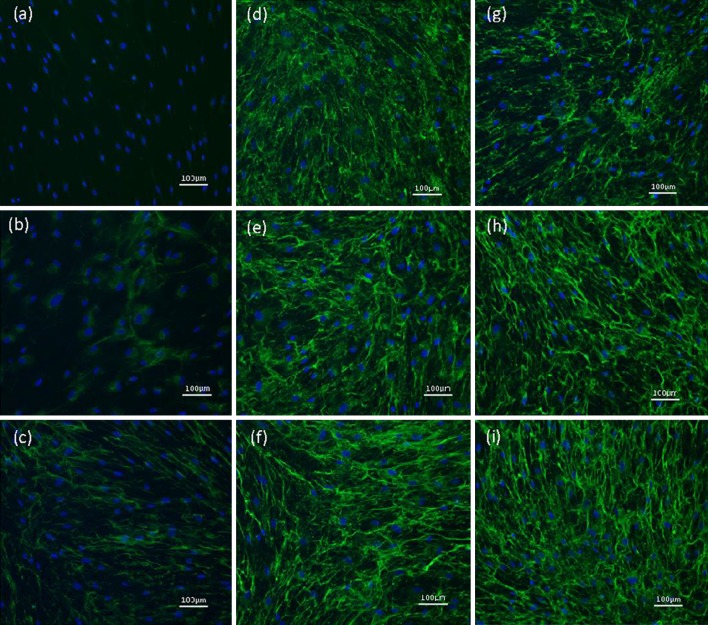
Effects of TGF-β1, macromolecular crowding, HGF and Botox type A on collagen-I biosynthesis analyzed by immunocytochemistry. (**a**) non-crowded control; (**b**) non-crowded + TGF-β1; (**c**) crowded control; (**d**) crowded + TGF-β1; (**e**) crowded + TGF-β1 + Botox 40 IU/ml; (**f**) crowded + TGF-β1 + Botox 80 IU/ml; (**g**) crowded + TGF-β1 + HGF 40 ng/ml; (**h**) crowded + TGF-β1 + HGF 100 ng/ml; (**i**) crowded + TGF-β1 + HGF 200 ng/ml

Standard conditions, as described above without antifibrotic agents were used to validate results from prior studies with rat cells in the current model utilizing human cells. Silver stain results for collagen deposition are shown in Fig. 1. In the non-crowded control group collagen-I-α was detected in the supernatant, but hardly in cell layer. After adding TGFβ-1 the signal increased in both fractions. This increase was more pronounced in the supernatant, and can be explained by the upregulated production of unprocessed collagen induced by TGFβ-1. In contrast, crowding resulted in an increased signal in cell layer and a decreased signal in the supernatant, reflecting the ability of crowding to enhance incorporation of collagen into the extracellular matrix. Finally, the combination of TGFβ-1 and crowding led to a significantly increased collagen deposition in cell layer (*p* = 0.016, Fig. 1b) and a decrease in supernatant, compared to the sole addition of TGFβ-1. Corresponding results were observed in immunocytochemistry (Fig. 2a–d).

**Fig. 3:**
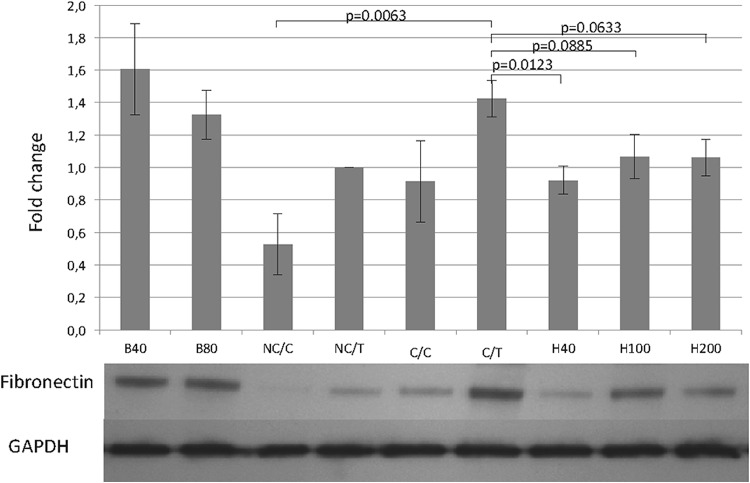
Effects of TGF-β1, macromolecular crowding, HGF and Botox type A on fibronectin analyzed by Western blot. NC/C = non-crowded control; C/C = crowded control; NC/T = non-crowded + TGF-β1; C/T = crowded + TGF-β1; H40 = crowded + TGF-β1 + HGF 40 ng/ml; H100 = crowded + TGF-β1 + HGF 100 ng/ml; H200 = crowded + TGF-β1 + HGF 200 ng/ml; B40 = crowded + TGF-β1 + Botox 40 IU/ml; B80 = crowded + TGF-β1 + Botox 80 IU/ml

Fibronectin was analyzed by western blot, revealing that separate addition of either TGFβ-1 or crowding molecules enhanced fibronectin deposition in the cell layer (Fig. 3). The combination of both resulted in a more pronounced upregulation and a statistically significant increase compared to the untreated group (C/T vs. NC/C; *p* = 0.0063).

**Fig. 4:**
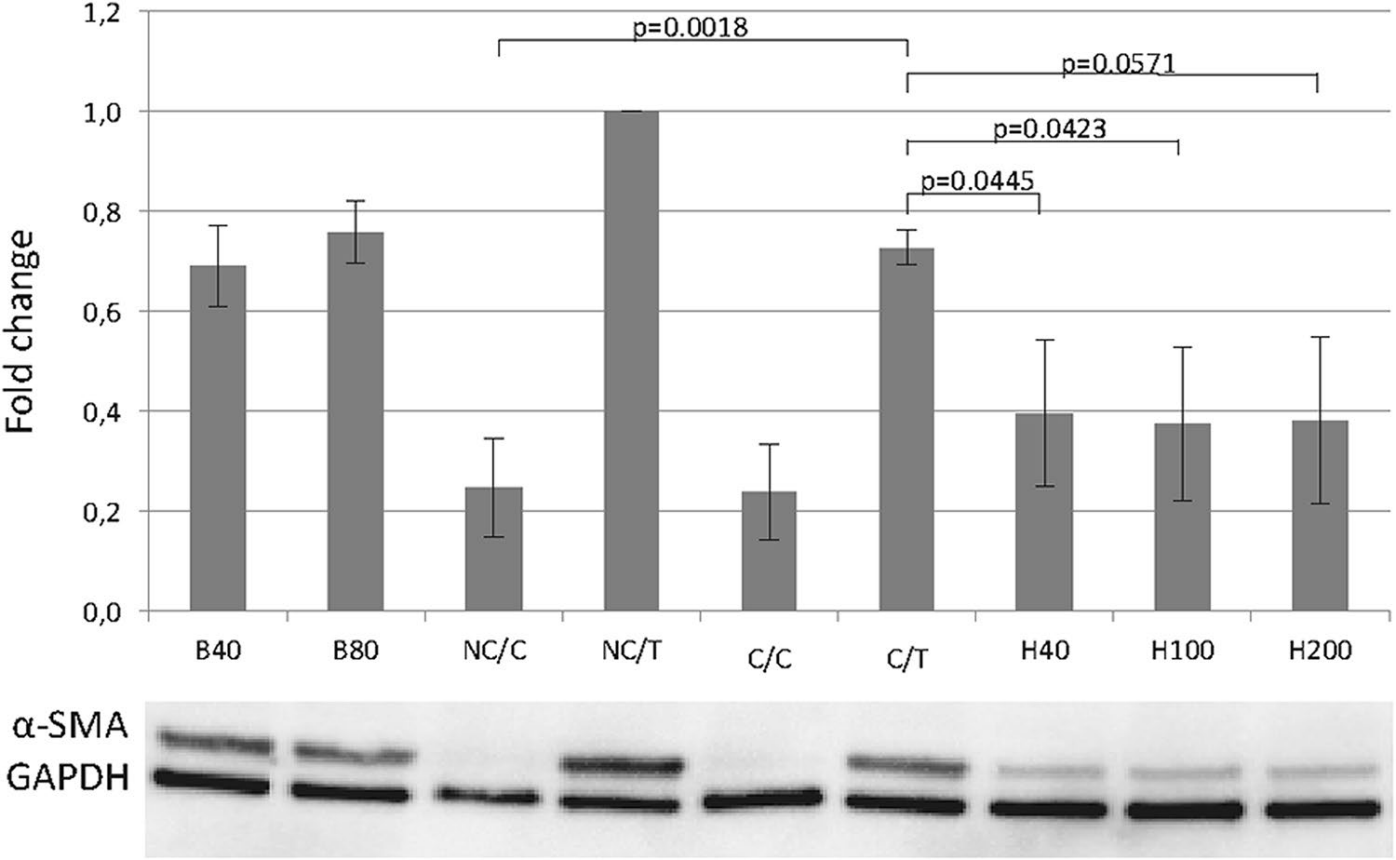
Effects of TGF-β1, macromolecular crowding, HGF and Botox type A on α-SMA (ACTA) analyzed by Western blot. NC/C = non-crowded control; C/C = crowded control; NC/T = non-crowded + TGF-β1; C/T = crowded + TGF-β1; H40 = crowded + TGF-β1 + HGF 40 ng/ml; H100 = crowded + TGF-β1 + HGF 100 ng/ml; H200 = crowded + TGF-β1 + HGF 200 ng/ml; B40 = crowded + TGF-β1 + Botox 40 IU/ml; B80 = crowded + TGF-β1 + Botox 80 IU/ml

Abundance of α-SMA was very low in cells cultured with standard medium or crowding alone (Fig. 4). Treating cells with TGFβ-1 induced high expression of α-SMA under standard and crowding conditions.

### Validation of antifibrotic agents

Addition of HGF at different concentrations showed a decrease for collagen α-bands in cell layer (Fig. 1), but compared to the crowded plus TGFβ-1 group this decline was only significant in the group treated with 100 ng/mL HGF (*p* = 0.035, see Fig. 1b). Results from immunocytochemistry are shown in Fig. 2g–i. To further investigate a potentially enhanced cell growth effect due to increasing HGF concentrations—which might counteract antifibrotic properties—cell nuclei were counted. We found a trend towards higher cell counts in chambers with high HGF concentrations; however, these results were not statistically significant (Supplementary Fig. 1).

HGF was capable of dampening fibronectin production (Fig. 3). Depending on the used concentration, the decline was most pronounced at 40 ng/mL (H40 vs. C/T; *p* = 0.0123) with nearly similar results for 100 and 200 ng/mL.

Expression of α-SMA could also be suppressed by HGF in a dose-dependent manner (see Fig. 4). Especially at 40 ng/mL (C/T vs. H40; *p* = 0.0445) and 100 ng/mL (C/T vs. H100; *p* = 0.0423) results were statistically significant.

BTXA did not show strong antifibrotic effects on collagen, fibronectin or α-SMA in any of the tested concentrations (40 or 80 IU/mL) (Figs. 2, 3, 4, supplementary Fig. 2–3).

## Discussion

A thorough in vivo exploration of VF fibrosis on the cellular level in humans is virtually impossible to achieve. Therefore, most studies have been investigating antifibrotic drugs in different animals or in vitro. In a recent paper, we described the successful establishment of an in vitro model of VF fibrogenesis based on the principles of MMC [9]. The current project employed human VFF and studied the effects of antifibrotic agents. In addition, a more comprehensive analysis was carried out by Silver stain, western blot and fluorescence microscopy. In accordance with previous papers in the field [6, 8], we found that crowding strongly supports the incorporation of collagen into the ECM, which is represented by the cell layer [14]. This is desirable for an in vitro model of fibrogenesis, because considerable amounts of (water-soluble) newly produced collagen in supernatants gets lost for analysis with every medium change under conventional culture conditions. ECM components were shown to be upregulated in the presented model, whereas the combination of TGF-β1 and macromolecules resulted in a further increase that exceeded the mere addition of single effects.

In 2010, Vyas et al. published data about a first in vitro model, using fibroblasts from normal human VF [12]. They used HGF in various concentrations to reverse the effects of TGF-β1. α-SMA expression of cells was evaluated with western blot. However, any effect on extracellular matrix components was not investigated in this study.

In a study by Kosinski et al. antifibrotic effects of dexamethasone were tested in immortalized human VF fibroblasts by quantitative polymerase chain reaction (qPCR) for mRNA of collagen [19]. Comparable methods were used by Suehiro et al. in a study with rat VF fibroblasts [20]. They investigated in vitro the impact of different concentrations of fibroblast growth factor 2 (bFGF) by qPCR for procollagen. Another interesting study by Kumai et al. investigated modulation of fibroblasts by stem cells [21]. In an in vitro trial, they co-cultured scar fibroblasts from vocal folds of rats with adipose-derived stem cells. Enzyme-linked immunosorbent assay (ELISA) was used to analyze supernatants. Cell proliferation and α-SMA were also evaluated. They found significant downregulation of collagen, cell proliferation and α-SMA with upregulation of hyaluronic acid.

Differences between fibroblasts from naïve and scarred rat vocal folds were analyzed in a recent paper by Kishimoto et al. [22]. They investigated collagen, α-SMA and tested for effects of HGF. However, antifibrotic properties of HGF were evaluated again at the transcriptome level and not at the level of mature proteins.

In conclusion, most in vitro trials on potential antifibrotic compounds evaluated collagen on the RNA level or measured procollagen with ELISA in supernatants. A proper investigation of mature collagen from a representative ECM has not been performed yet. Promising results have been published with the used methods but only with respect to effects of antifibrotic agents on secretion and/or biosynthesis of collagen precursors. Evaluation of agents that interfere with later steps of collagen formation like any inhibitor of procollagen C-proteinase would gain misleading results with the proposed methods. In contrast, our in vitro model assures sufficient in vitro production of the mature extracellular matrix to assess compounds that interfere with any step in the formation of collagen.

In the current study HGF and Botox were tested. HGF is reported to have significant effects in treating fibrosis of various organs [15, 16, 23, 24]. In VF scarring, in vitro studies have shown that HGF increased mRNA expression of hyaluronic acid synthases. Animal studies (canine model) proved that local administration of HGF lead to an improvement of viscoelastic properties, and decreased collagen levels [25, 26].

In another canine study, HGF was injected intracordally 1 month after VF stripping. The applied dose of HGF (500 ng/0.5 mL PBS) led to significantly improved patterns in vibration analysis. Mizuta et al. tested pharmacokinetics and tissue response to HGF in rats, showing a rapid decrease of local HGF concentration after intracordal injection. Furthermore HGF was hardly detected in blood after local injection in their study [27].

Only few studies have addressed the optimum dose of HGF. Suehiro et al. studied the effects of HGF application in aged rats, reporting beneficial effects when using a dose of 10 ng/µL [28]. Noteworthy doses used in this and other in vivo experiments mentioned above exceeded the doses in our in vitro model on average by a factor of 100. On the one hand, the high range of administered doses can be explained at least partially by the inherent differences of in vitro and in vivo experiments. On the other hand, one must state, however, that aged VF differs significantly from scarred VF.

In our in vitro model, we could confirm that HGF reduced the amount of collagen significantly at a concentration of 100 ng/mL. However, a higher concentration of HGF (200 ng/mL) did not lead to antifibrotic effects in our experiments, whereas in the lowest concentration (40 ng/mL) there was a trend towards decreased collagen. Since a cell growth effect of HGF has been described in human pancreatic cancer cell lines [29] we performed cell count analysis but could not confirm a significant boost in cell proliferation.

Simultaneously we could observe a dose-dependent effect between HGF and α-SMA even at higher doses which puts our findings in line with previous studies in other contexts [30]. Noteworthy, results concerning α-SMA did not differ substantially between TGF-β1 stimulation alone, or crowding plus TGF-β1 stimulation which shows that effects of crowding on myofibroblast differentiation are limited and the combination of both agents (TGF and macromolecules) seem necessary to build a representative in vitro model.

We also tested for antifibrotic effects of BTXA, which has not been addressed so far in the in vitro setting. Of note, BTXA is widely used to treat hypertrophic scars in daily clinical routine. Studies about this topic also showed measurable effects of BTXA in treatment of scars in an animal model and in humans [31, 32]. However, in our VFF in vitro experiments, different concentrations of BTXA did not lead to significant changes in collagen, fibronectin, and α-SMA. It thus remains uncertain whether beneficial effects of BTXA on wound healing also comprise a cell-autonomous effect on resident fibroblasts, in addition to its action on the neuromuscular axis.

## Conclusion

A great variety of different animal models (rats, dogs, rabbits) and numerous settings (treatment right after injury [33] vs. in early [34]/ late [25] scar, stimulation of non-scarred cells) [28, 35, 36] hamper the development of effective strategies in laryngeal tissue engineering. Our in vitro fibrogenesis model based on the principles of MMC might contribute substantially to a standardization of experiments in laryngology. The approach presented in this study will allow a fast screening procedure of potential antifibrotic agents foreseen for treatment of laryngeal scar and might reduce in vivo animal trials significantly. Moreover, the application of our in vitro fibrogenesis model in extensive high-throughput screens, e.g. using libraries of chemical compounds, appears as a logical next step able to foster the discovery of novel antifibrotic compounds.

## Electronic supplementary material

Below is the link to the electronic supplementary material.

Supplementary material 1 (PDF 307 KB)
