# Supplementary material for: Towards an in vitro fibrogenesis model of human vocal fold scarring
Source: Eur Arch Otorhinolaryngol. 2018 Mar 8;275(5):1211–8. doi: 10.1007/s00405-018-4922-7 (PMC5893733; doi:10.1007/s00405-018-4922-7)
Supplement: Supplementary file 1 — Supplementary material 1 (PDF 307 KB) [file 405_2018_4922_MOESM1_ESM.pdf]

ELECTRONIC SUPPLEMENTARY MATERIAL

European Archives of Oto-Rhino-Laryngology

Towards an *in vitro* fibrogenesis model of human vocal fold scarring

M. Graupp, M.D., D.MSci[1], B. Rinner[2], Ph.D.[2], M.T. Frisch, MSc[2], G. Weiss, Ph.D.[3], J. Fuchs, MSc[3], M. Sundl[3], A. El-Heliebi, Ph.D.[3], G. Moser, Ph.D.[3], L.P. Kamolz, M.D.[4], M. Karbiener, Ph.D.[1#], M. Gugatschka[1], M.D., D.MSci

1. Department of Phoniatrics, ENT University Hospital Graz, Medical University of Graz, Austria.
2. Division of Biomedical Research, Core Facility Alternative Biomodels and Preclinical Imaging, Medical University of Graz, Austria
3. Institute of Cell Biology, Histology and Embryology, Medical University of Graz, Austria
4. Division of Plastic, Aesthetic and Reconstructive Surgery, Department of Surgery, Medical University of Graz, Austria

[#]Corresponding author

E-mail: michael.karbiener@medunigraz.at. Phone: +43-316-385-30083

ENT University Hospital Graz, Dept. of Phoniatrics, Medical University of Graz, Auenbruggerplatz 26, 8036 Graz, Austria.

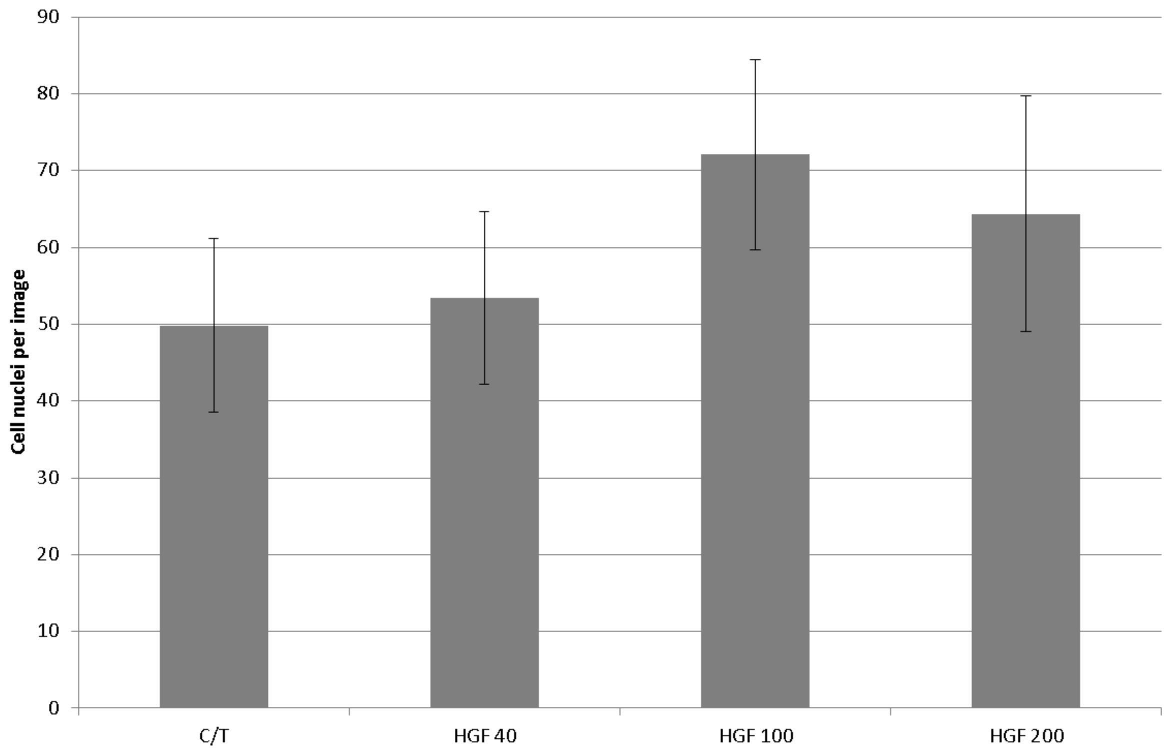

Supplementary Figure 1: HGF promotes hVFF proliferation. DAPI-stained nuclei were counted 5 days after HGF treatment; C/T=crowded+TGF-β1; H40=crowded+TGF-β1+HGF40ng/ml; H100=crowded+TGF-β1+HGF100ng/ml; H200=crowded+TGF-β1+HGF 200ng/ml;

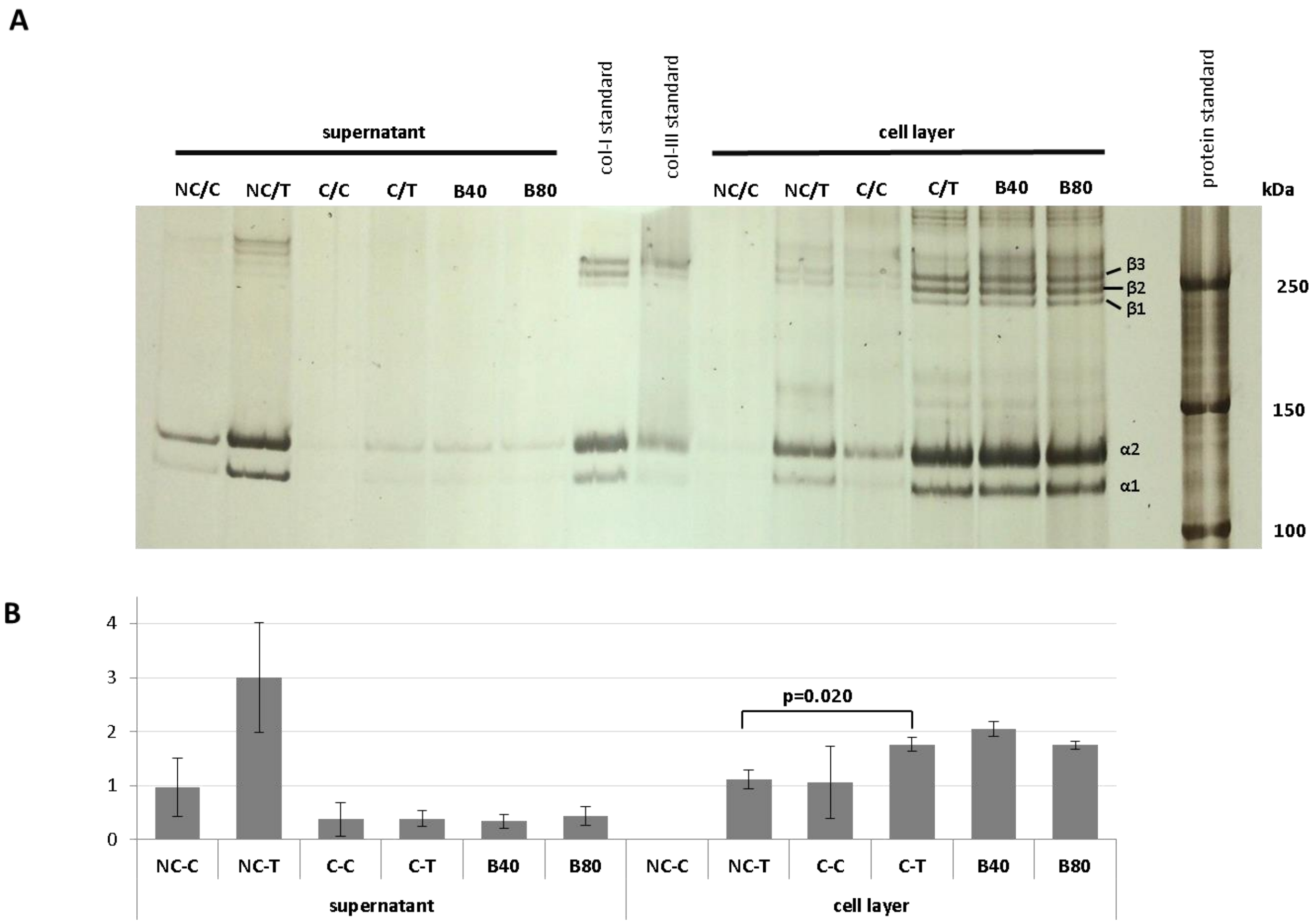

Supplementary Figure 2: Effects of TGF-β1, macromolecular crowding and Botox type A on collagen biosynthesis analyzed by silver stain. (A) Image of silver stained gel showing results for collagen deposition in standard conditions and with Botox type A treatment; NC/C=non-crowded control; C/C=crowded control; NC/T=non-crowded+TGF-β1; C/T=crowded+TGF-β1; B40=crowded+TGF-β1+Botox40IU/ml; B80=crowded+TGF-β1+Botox80IU/ml; (B) Densitometric analysis of silver stain for collagen deposition; condition NC/C of cell layer served as reference.

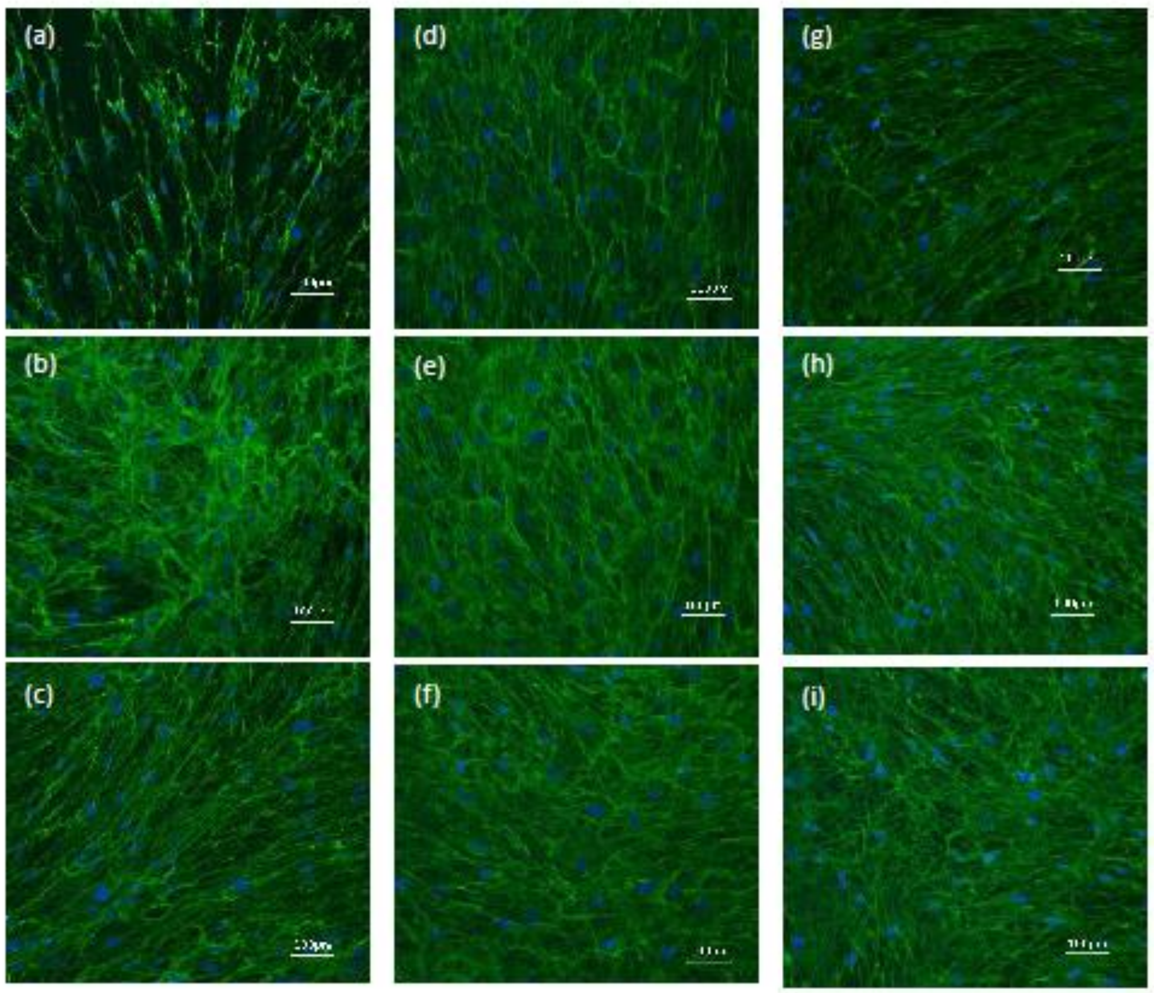

Supplementary Figure 3: Effects of TGF-β1, macromolecular crowding, HGF and Botox type A on fibronectin. Immunocytochemistry for fibronectin in standard conditions and after treatment with Botox or HGF; (a) non-crowded control; (b) non-crowded+TGF-β1; (c) crowded control; (d) crowded+TGF-β1; (e) crowded+TGF-β1+Botox40IU/ml; (f) crowded+TGF-β1+Botox80IU/ml; (g) crowded+TGF-β1+HGF40ng/ml; (h) crowded+TGF-β1+HGF100ng/ml; (i) crowded+TGF-β1+HGF200ng/ml.

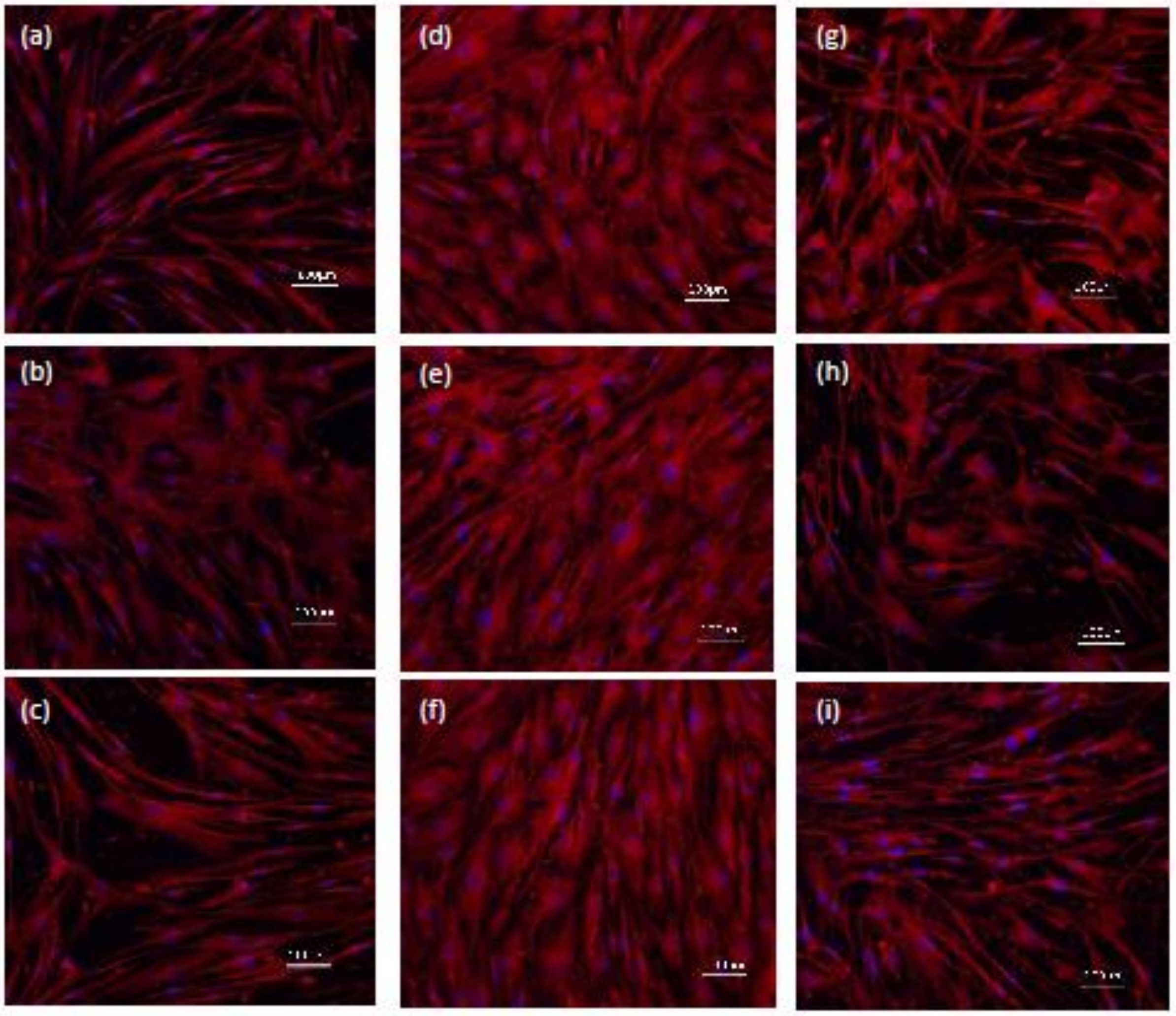

Supplementary Figure 4: Effects of TGF-β1, macromolecular crowding, HGF and Botox type A on α-SMA. Immunocytochemistry for α-SMA in standard conditions and after treatment with Botox or HGF; (a) non-crowded control; (b) non-crowded+TGF-β1; (c) crowded control; (d) crowded+TGF-β1; (e) crowded+TGF-β1+Botox40IU/ml; (f) crowded+TGF-β1+Botox80IU/ml; (g) crowded+TGF-β1+HGF40ng/ml; (h) crowded+TGF-β1+HGF100ng/ml; (i) crowded+TGF-β1+HGF200ng/ml.
